# Hypoglycaemic activity of *Bauhinia holophylla* through GSK3-β inhibition and glycogenesis activation

**DOI:** 10.1080/13880209.2019.1599962

**Published:** 2019-04-22

**Authors:** Nathalia Ap. De Paula Camaforte, Luiz Leonardo Saldanha, Priscilla Maria Ponce Vareda, João M. Rezende-Neto, Mario R. Senger, Aislan Q. Delgado, Henrique J. N. Morgan, Natalia Moretti Violato, Laís Goyos Pieroni, Anne Lígia Dokkedal, Floriano P. Silva-Júnior, José Roberto Bosqueiro

**Affiliations:** a Institute of Biosciences, São Paulo State University, Botucatu, São Paulo, Brazil;; b Laboratory of Experimental and Computational Biochemistry of Drugs, Oswaldo Cruz Institute (FIOCRUZ), Rio de Janeiro, Brazil;; c Department of Biological Sciences, São Paulo State University, Bauru, São Paulo, Brazil;; d Department of Physical Education, São Paulo State University, Bauru, São Paulo, Brazil

**Keywords:** Antidiabetic, Fabaceae, flavonoid-*O*-glycosides, liver, plasma lipids

## Abstract

**Context:***Bauhinia* L. species, including *Bauhinia holophylla* (Bong.) Steud. (Fabaceae), have traditionally been used to treat diabetes. *Bauhinia* is a complex botanical genus, and the indiscriminate use of the diverse *Bauhinia* species is reflected in the experimental divergence of their medicinal potential.

**Objective:** The hypoglycaemic and hypolipidaemic effects, molecular mechanism of action and phytochemical properties of an authentic extract of *B. holophylla* leaves were evaluated.

**Materials and methods:** A phytochemical study of a 70% EtOH extract was performed using FIA-ESI-IT-MS/MS^n^ and HPLC-PAD-ESI-IT-MS. The extract (200 or 400 mg/kg b.w.) was administered for 14 days to streptozotocin-induced diabetic Swiss mice. Glucose tolerance and insulin sensitivity, blood parameters, gene and protein expression, and the *in vivo* and *in vitro* inhibition of intestinal glucosidases were assessed.

**Results:** HPLC-PAD-ESI-IT-MS analysis identified flavonoid derivatives of quercetin, myricetin, luteolin and kaempferol. Treatment with 400 mg/kg of the extract reduced blood glucose (269.0 ± 32.4 mg/dL vs. 468.0 ± 32.2 mg/dL for diabetic animals), improved glucose tolerance, decreased cholesterol and triglyceride levels, and increased the mRNA expression of proteins involved in glucogenesis in the liver and muscle, such as PI3-K/Akt, GS, GSK3-β (ser-9), AMPK and Glut4. The activity of intestinal maltase was inhibited *in vitro* (IC_50_: 43.0 µg/mL for the extract compared to 516.4 µg/mL for acarbose) and *in vivo*.

**Discussion and conclusions:** Treatment with *B. holophylla* was associated with a marked hypoglycaemic effect through the stimulation of glycogenesis and inhibition of gluconeogenesis and intestinal glucose absorption, without increasing basal insulinaemia.

## Introduction

*Bauhinia* L. *s.l.* (Fabaceae) is a large genus with about 340 species in the tribe Cercideae Bronn with a pantropical intercontinental disjunct distribution (Legume Phylogeny Working Group-LPWG 2013; Lin et al. 2014). These species are popularly known as ‘cow's foot’, ‘cow's paw’, or *pata-de-vaca* due to their characteristic bilobate or bifoliolate leaves with pulvinate petiole and basal actinodromous or acrodromous venation. The leaf architecture characteristics of this genus have been extensively studied and form the basis of the biogeographic history, identification and classification of *Bauhinia s.l.* (Lin et al. 2015; Fortunato et al. 2017). Several studies have suggested new organizations of this large genus, and historical factors have complicated its taxonomy and nomenclature (Wunderlin et al. 1987; Lewis and Forest 2005; Wunderling 2010). Recent molecular phylogenetic revisions have revealed that *Bauhinia s.l*. is paraphyletic and may be split into nine separate genera including *Bauhinia* L. *s.s.*, *Gigasiphon* Drake, *Tylosema* (Schweinf.) Torre et Hillc., *Barklya* F. Muell., *Lysiphyllum* (Benth.) de Wit, *Phanera* Lour., *Lasiobema* (Korth.) Miq., *Piliostigma* Hochst., and *Schnella* Raddi (Hao et al. 2003; Sinou et al. 2009; Wunderlin 2010).

Ethnopharmacological studies have highlighted several *Bauhinia s.l*. species frequently used as herbal products in folk medicine to treat different types of pathology, such as pain, inflammation, infections and, particularly, diabetes (Cechinel et al. 2009). Species including *Bauhinia forficata* Link (Lino et al. 2004), *B. cheilantha* (Bong.) Steud. (Almeida et al. 2006), *B. candicans* Benth (Fuentes et al. 2004), *B. monandra* Kurz (Menezes et al. 2007), and *B. megalandra* Griseb (Gonzalez-Mujica et al. 2003) have exhibited antidiabetic properties in mice and rats, demonstrating the potential of *Bauhinia* as a significant source of bioactive metabolites. Recently, Rozza et al. (2015) showed that an extract of the leaves of *Bauhinia holophylla* (Bong.) Steud., a native shrub from the Brazilian Cerrado, has antiulcer activity in rats through its antioxidant and anti-inflammatory properties.

Phytochemical studies have identified a variety of flavonoids (Silva and Cechinel-Filho 2002), with flavonols representing the most abundant subclass, followed by flavones, flavans and flavanones (Farag et al. 2015).

However, despite the extensive phytochemical characterization of *Bauhinia* extracts and the confirmation of its antidiabetic properties (Juliant 1931; Pepato et al. 2002; Silva et al. 2002; Silva and Cechinel-Filho 2002), there are some contradictory results in the literature regarding the antidiabetic potential of certain species (Almeida and Agra 1986; Volpato et al. 1999; Silva et al. 2002; Damasceno et al. 2004; Pinheiro et al. 2017). For example, a recent study evaluating the hypoglycaemic potential of *B. holophylla*, a species used in folk medicine to treat diabetes (Oliveira and Saito 1989), failed to demonstrate antidiabetic activity (Pinheiro et al. 2017).

In general, the characteristic bilobed or bifoliolate leaves are used to recognize these species, leading to wide and indiscriminate use of the leaves of any *Bauhinia* species as hypoglycaemic agents (Salatino et al. 1999; Fortunato et al. 2017). The correct identification of species is difficult, and inaccuracies can cause misidentification of species, resulting in reduced effectiveness of the extracts (Ferreres et al. 2012). In view of the ethnopharmacological indications, as well as the chemical constitution of the genus *Bauhinia*, we hypothesize that the 70% EtOH extract of *B. holophylla* is able to reduce the glycaemia of diabetic animals. Therefore, this study comprehensively investigated the potential hypoglycaemic effects and mechanisms of action of an authenticated extract of *B. holophylla* leaves, using HPLC-PAD-ESI-IT-MS to establish the chemical profile of the extract, to advance the knowledge regarding the use of *Bauhinia* extracts and their efficacious and safe use in phytotherapy.

## Materials and methods

### Plant material and extraction

Samples of *B. holophylla* leaves were collected in November 2010 at the Jardim Botânico Municipal de Bauru (22°20′30ʺ S, 49°00′30ʺ W), SP, Brazil. Voucher specimens were prepared and identified by Prof. Dr. Ângela Maria Studart da Fonseca Vaz and stored at the Herbarium of the Jardim Botânico do Rio de Janeiro (Rio de Janeiro, RJ, Brazil) under code number RB 507.043.

Fresh leaves were hot air-dried at 45 °C for 48 h. The separated powdered leaves (220 g) were extracted with ethanol and water (EtOH–H_2_O, 7:3, v/v) by percolation at room temperature. The hydroethanolic solution was filtered and concentrated to dryness under reduced pressure at 40 °C, yielding 65 g (29.5%) of the hydroethanolic extract (70% EtOH).

### Flow injection analysis with electrospray ionization and an ion trap analyzer coupled with a mass detector (FIA-ESI-IT-MS/MS^n^) and high-performance liquid chromatography coupled to a photodiode array and mass spectrometer detector (HPLC-PAD-ESI-IT-MS) analysis instrumentation

The chromatographic profile of the *B. holophylla* 70% hydroethanolic extract was performed using an Accela High-Speed LC (Thermo Scientific®, San Jose, CA), Luna C_18_ column (250 × 4.6 mm i.d.; 5 μm) (Phenomenex^®^ Inc., Torrance, CA) coupled to a photodiode array detector (PAD) (Accela PDA detector, Thermo Scientific^®^) and LCQ Fleet with 3 D Ion Trap (IT) and ionization by electrospray (ESI). The mobile phase was methanol (eluent A) and ultrapure water (eluent B), both containing 0.1% formic acid. The ratio was 0–15 min with 25–40% A, 15–30 min with 40–55% A and 30–40 min with 100% A. The injection volume was 20.0 μL; the column temperature was 25 °C; the flow ratio was 1 mL/min, and the chromatogram was recorded at 350 nm. The effluent from the HPLC was directed into the ESI probe.

Using this method, we determined the most intense parent ion for each peak in the chromatogram. A second event, in the negative ion mode, was performed using the same equipment described above. The 70% hydroethanolic extract was dissolved in MeOH–H_2_O (8:2% v/v) and infused in the ESI source by flow injection analysis (FIA) using a syringe pump; the flow rate was 33 μL/min. The capillary voltage was −20 V, the spray voltage was 4 kV, and the tube lens offset was −55 V. The capillary temperature was 275 °C. Nitrogen was used both as the drying gas at a flow rate of 60 (arbitrary units) and as the nebulizing gas. The nebulizer temperature was 280 °C, and the potential was −4 V for the capillary. Negative ion mass spectra were recorded in the *m/z* range of 100–1550. Scan events were prescribed to run in the LCQ mass spectrometry. The first event was a full-scan spectrum to acquire data on the deprotonated compounds within the scan range established. The second scan event was an MS/MS experiment performed using a data-dependent scan on the deprotonated molecule [M-H]^−^. The collision energy for MS/MS was adjusted to 10–25%. These analyses were conducted according to the method of Saldanha et al. (2013).

### Ethics statement

All the experiments were conducted according to a protocol that was submitted and approved by the Animal Experimentation Ethics Committee of UNESP/Araçatuba, SP, Brazil (Process n° 01742-2012).

### Animals

Male Swiss mice (aged 60 days, weighing 40 g) were obtained from São Paulo State University, São Paulo, Brazil. The animals were kept under standard environmental conditions (22 ± 2 °C), with a 12 h dark/light cycle. Animals were fed with industrialized food (Labina^®^, Purina, Brazil), and water was given *ad libitum*.

### Acute toxicity test

Normoglycaemic mice were divided into 2 groups (*n* = 10) (CTL and BH) and fasted for 4 h; after this period, the animals received saline (1 mL/kg) or an extract of *B. holophylla* (2 g/kg b.w.) by gavage. The animals had their behaviour analyzed at 30, 60, 90, 120, 240 and 360 min after the gavage according to the Hippocratic screening described by Brito (1994). Next, the animals were observed and weighed for the following 14 days. At the end of this period, the animals were sacrificed, and the liver, kidneys, lungs, heart and spleen were collected, weighted and the relative weights were calculated.

### Induction of experimental diabetes

The diabetes induction was performed using a single injection of 150 mg/kg b.w. of streptozotocin (STZ – Sigma-Aldrich^®^, St. Louis, MO) dissolved in 0.1 mL of citrate buffer 0.1 M (pH 4.5) and immediately injected intraperitoneally into mice fasted for 12–14 h (Rakieten et al. 1963). The animals were kept fasted for 3 h after induction and for the next 24 h received a 10% glucose solution to prevent hypoglycaemia. On the 7th day post-STZ-injection, the animals with fasting glycaemia levels higher than 250 mg/dL were included in the study.

### Experimental design

The animals were randomly divided into three sets of four to seven groups (*n* = 8/group): CTLSAL – normoglycaemic control mice treated with saline; CTLEXT200 – normoglycaemic control mice treated with the extract of *B. holophylla*, at 200 mg/kg b.w.; CTLEXT400 – normoglycaemic control mice treated with the extract of *B. holophylla*, at 400 mg/kg b.w.; STZSAL – diabetic mice treated with saline; STZMET – diabetic mice treated with metformin, at 300 mg/kg b.w.; STZEXT200 – diabetic mice treated with the extract of *B. holophylla*, at 200 mg/kg b.w.; and STZEXT400 – diabetic mice treated with the extract of *B. holophylla*, at 400 mg/kg b.w. The saline, extract and metformin were administered orally by gavage once a day for 14 consecutive days. The selected doses (200 and 400 mg/kg) of *B. holophylla* extract was based on studies in the literature with other species of *Bauhinia* (Lino et al. 2004; Kumar et al. 2012). The lyophilized extract was dissolved in fresh saline solution (0.9% NaCl, pH 7.4) prior to the gavage.

### Glycaemia, food and water intake, and body weight

For food intake measurement, the remaining chow after a 24 h period was normalized to the total body mass from each cage. Body mass was measured daily from the beginning of the treatment until the day of euthanasia using a conventional electronic balance (Tecnal, Piracicaba, Brazil). On the last day of treatment, the groups of fasted (8–10 h) mice had blood collected from the tail to measure blood glucose levels with a glucometer (One Touch, Johnson & Johnson, NJ). Immediately after blood collection, the mice were euthanized (exposure to CO_2_ followed by decapitation), and the trunk blood was collected in EDTA-NaF-containing tubes (Glistab – Labtest, Lagoa Santa, MG, Brazil) to obtain the plasma. The plasma, obtained after blood centrifugation (600 *g*), was stored at −80 °C in several aliquots for posterior measurements.

### Intraperitoneal glucose tolerance test (ipGTT)

On the 14th day of treatment, the animals from groups CTLSAL, CTLEXT400, STZSAL, STZEXT400 and STZMET were fasted for 8–10 h, and the fasting blood glucose was measured and defined as time 0. A glucose load (2 g/kg b.w.) was injected intraperitoneally after 30 min of extract, saline or metformin administrations, and then, the blood glucose was measured at 15, 30, 60, 90 and 120 min after glucose administration (Rafacho et al. 2008). Blood samples were obtained from the tail tip under anaesthesia (Tiopental^®^, 60 mg/kg b.w.), and glucose levels were measured using an enzymatic kit (Dolles^®^, Goiás, Brasil). The groups treated with 200 mg/kg were not submitted to IPGTT because, as will be demonstrated in the results section, there was no significant decrease in fasting glycaemia with this dose.

### Biochemical parameters

At the end of the treatment, the animals were fasted for 8–10 h, and blood samples were collected and centrifuged at 1500 rpm for 10 min to obtain serum and then were stored at −80 °C. Total cholesterol (TC), triglycerides (TGs) and total proteins were measured by commercial kits (Dolles^®^, Goiás, Brasil). The homeostasis model assessment of insulin resistance (HOMA-IR) was calculated using glucose and insulin concentrations according to the following formula: fasting blood glucose (mg/dL)×fasting insulin (pmol/L) (Matthews et al. 1985). Basal plasma insulin content was measured using a rat/mouse insulin ELISA kit (Millipore^®^, St. Charles, MO; #EZRMI-13K).

### Hepatic and muscular glycogen

The animals were sacrificed on the 14th day, after 8–10 h of fasting. The glycogen content was measured as previously described by Rafacho et al. (2008). Briefly, liver and muscle samples (300–500 mg) were stored in tubes containing 30% KOH (Mallinckrodt Baker^®^, Paris, France) and boiled for 1 h until completely homogenized. Na_2_SO_4_ (Mallinckrodt Baker^®^, Paris, France) was then added, and glycogen was precipitated with 100% ethanol. The samples were centrifuged at 800 *g* for 10 min, the supernatants were discarded, and the glycogen was dissolved in hot distilled water. Ethanol was added, and the pellets were obtained after a second centrifugation. The pellets were then dissolved in distilled water to a final volume of 20 mL. The glycogen content was measured by treating a fixed volume of each sample with phenol reagent and H_2_SO_4_, and the absorbance was read at 490 nm with a spectrophotometer (BioTek^®^ PowerWave XS).

### RNA isolation and reverse real-time transcription-polymerase chain reaction (qRT-PCR)

Total mRNA was isolated from the mouse liver according to a TRIzol extraction protocol. After RNA quantification by Nanodrop (Thermo Scientific^®^) and Qubit (Invitrogen^®^), reverse transcription into cDNA was performed using a QuantiTect Reverse Transcription Kit (Qiagen^®^) according to the manufacturer’s instructions. qRT-PCR was used to analyze the gene expression using a TaqMan Gene Expression MasterMix (Applied Biosystems^®^). The reaction was performed using 96-well plates with a final volume of 5 µL, which included 1 µL of cDNA and 0.25 µL of each pre-manufactured gene sequence (PI3Kp85α-Mm00803160_m1; AKT1/2-Mm01331626_m1; GSK3β-Mm00444911_m1; glycogen synthase 2-Mm01267381_m1; G6Paseα-Mm00839363_m1; GLUT4-Mm01245502_m1; PEPCK1-Mm01247058_m1; glycogen phosphorylase-Mm01289790_m1; GAPDH-Mm03302249_g1) (TaqMan Gene Expression Assay, Applied Biosystems^®^), 2.5 µL of the TaqMan Gene Expression MasterMix (Applied Biosystems^®^) and 1.25 µL of RNase-free water. The reaction was performed in 40 cycles of StepOnePlus (Applied Biosystems^®^). The data obtained were analyzed using the relative quantification method of gene expression (ΔΔCt). GAPDH was used as the endogenous control (Calegari et al. 2012).

### Protein expression by Western blotting

Fragments of liver obtained from fed animals were homogenized in cell lysis buffer (Cell Signalling^®^, Beverly, MA) using a Polytron PT 1200 C homogenizer (Brinkmann Instruments^®^, NY) and subsequently sonicated (Fisher Scientific^®^, Suwanee, GA) (2 pulses of 15 s) at an intermediate speed. Protein concentrations were measured using the Bradford method, according to the manufacturer’s instructions (Bio-Rad Laboratories^®^, Hercules, CA). Aliquots (80 µg) were boiled at 100 °C for 4 min in 30% of the volume in Laemmli buffer. The samples were fractioned on an electrophoresis system (Mini-Protean II, Bio-Rad^®^, Hercules, CA) in a polyacrylamide gel with the appropriate pore size according to the molecular weight. Subsequently, the proteins were transferred to nitrocellulose membranes (Bio-Rad^®^, Hercules, CA) in the presence of 20% methanol and 0.02% SDS at a constant voltage of 120 volts. The membrane was blocked with 5% BSA basal solution (10 mM Trizma Base, 150 mM NaCl, 0.05% Tween 20) for 1 min. Next, each membrane was washed and incubated for 10 min at room temperature with one of the following appropriate primary polyclonal antibodies from Santa Cruz Biotechnology^®^: anti-Akt (sc-1619, 1:1000 dilution), anti-pAkt (sc-16646, 1:350 dilution), anti-PI3K-p85α (sc-56938, 1:1000 dilution), anti-pPI3K-p85α (sc-12929, 1:800 dilution), anti-GS (sc-99029, 1:500 dilution), anti-pGSK3β (sc-81494, 1:500 dilution), anti-G6Pase (sc-27198, 1:500 dilution), anti-PEPCK (sc-32879, 1:500 dilution), anti-AMPK (sc-74461, 1:800 dilution), anti-GLUT4 (sc-1608, 1:500 dilution), anti-PTP1B (sc-1718, 1:250 dilution), anti-GAPDH (sc-25778, 1:1000 dilution) and anti-β-actin (sc-130656, 1:1000 dilution) using SNAP i.d. (Millipore^®^, St. Charles, MO). After washing in TBST, the membranes were incubated with the appropriate secondary antibody (Santa Cruz Biotechnology^®^, CA). The membranes were washed and incubated in a dark room with a luminal chemiluminescent substrate (Pierce^®^, Rockford, IL) and exposed to an auto-radiographic film (Kodak T-Mat G/RA, Rochester, NY). The intensities of the bands were quantified by densitometry (Epson Expression 1600, Long Beach, CA). The bands were first normalized by β-actin or GAPDH, and then the relation between phosphorylated/total forms was calculated for each protein (Rafacho et al. 2009).

### Enzymatic kinetic assays for α-amylase and α-glucosidase

The enzymatic activities of amylase and glucosidases were evaluated in a final volume of 50 μL in 384-well microplates, adapted from previous studies (Ferreira et al. 2010; Gonzaga et al. 2014). The reaction mixture for yeast maltase containing potassium phosphate buffer (50 mM, pH 7.0) was preincubated at 37 °C for 5 min and the reaction was initiated by PNP-G (1 mM) addition. The reaction mixture for porcine pancreatic α-amylase containing HEPES buffer (50 mM, pH 7.0), CaCl_2_ (5 mM), NaCl (100 mM) was preincubated at 37 °C for 5 min and the reaction was initiated by the addition of CNPG3 (1 mM). Absorbance reading was performed on an automated microplate reader (Molecular Devices, Sunnyvale, CA), where *p*-nitrophenol appearance rate was measured at 405 nm wavelength. The initial velocity was calculated by linear regression of the slope from the linear portion of the reaction progress curves. Concentrations of DMSO up to 1% were used in the assays and not significantly affect enzymatic activities.

### IC_50_ determination and reversibility binding assays

Reactions were performed as described above. The extract concentration which promotes 50% inhibition of enzymatic activities (IC_50_) were determined by titrating at least eight concentrations of crude extracts ranging from 0.08 to 1250 μg/mL, together with each enzyme for 5 min at 37 °C before the reactions. IC_50_ values were determined in Sigmaplot 12.0 software (Systat Software Inc, USA) by adjusting the residual activity data and extract concentrations to the logarithmic equation of 4 parameters: Activ. Res.=Min+(max-min)/{(1+([I]/IC_50_) (−CoF × Hill)}. Acarbose was used as a positive control for potency comparison.

For reversibility binding assays, both glucosidases were incubated at a concentration of 100-fold of the required value for activity assay with an inhibitor concentration equivalent to 10-fold IC_50_ (Copeland 2005; Senger et al. 2012). After 30 min of incubation, the reaction was started by 100-fold dilution in the reaction buffer containing the substrate. The progress curve was compared to a similar sample of the enzyme incubated and diluted in the absence of the inhibitor.

### Starch loading in normal mice

Normal mice were kept fasted during 10–12 h and then separated in three groups CTL (treated with saline), AC (treated with Acarbose 10 mg/kg) and BH (treated with *B. holophylla* extract 400 mg/kg b.w.). Blood samples were collected at time zero and then animals received their respective treatments. After 10 min, a starch solution (2 g/kg) was loaded. After gavage, the glycaemia was measured at times 30, 60 and 120 min using glucometer (OneTouch, Johnson & Johnson). Deviation in blood glucose concentration from the basal value was analyzed and represented as delta blood glucose.

### Maltose loading in normal mice

Normal mice were kept fasted during 10–12 h and then separated in CTL (treated with saline), AC (treated with acarbose 10 mg/kg) and BH (treated with *B. holophylla* extract 400 mg/kg b.w.). Blood samples were collected at time 0 and then animals received their respective treatments; 10 min after, a maltose solution (2 g/kg) was loaded. After gavage, the glycaemia was measured at 30, 60 and 120 min using glucometer (OneTouch, Johnson & Johnson^®^). Deviation in blood glucose concentration from the basal value was analyzed and represented as delta blood glucose.

### Statistical analysis

The results are expressed as means ± standard error of the means (S.E.M.). For multiple comparisons, ANOVA was used, followed by Tukeyʼs *post-hoc* test; for comparisons between two groups, Studentʼs *t*-test was used. The significance level adopted was *p* < 0.05.

## Results and discussion

### Phytochemical characterization

Metabolite profiling of the extract of leaves of *B. holophylla* using flow injection analysis with electrospray ionization and an ion trap analyzer with a mass detector (FIA-ESI-IT-MS/MS^n^) as well as high-performance liquid chromatography coupled to a photodiode array and a mass spectrometer detector (HPLC-PAD-ESI-IT-MS) identified 13 flavonoids. The constituents were tentatively identified based on their retention (Rt), ultraviolet (UV) and mass spectrometer (MS) data in comparison with flavonoid fragmentation patterns previously described by Saldanha et al. (2013). Data for the identification of all peaks present in the analytical chromatogram are shown in Table 1.

**Table 1.** HPLC-PAD-MS data [Retention time (Rt), ultraviolet (UV) and detected ions (LC-MS ions)] and MS/MS^n^ data of compounds identified in *Bauhinia holophylla*.

Peak (ID)	Compound	Rt (min)	UV-vis (*λ*_max_/nm_._)	LC-MS ions [M-H]^−^	MS/MS^n^	Tentatively assignments
1	myricetin-*O*-hexoside	13.83	358/308^sh^/263	479.3	958, 316.7	UV/MS
2	myricetin-*O*-pentoside	16.51	363/268	449.1	898.5, 316.9	UV/MS
3	myricetin-*O*-deoxyhexoside	17.43	352/256	463.8	316.5	UV/MS
4	quercetin-*O*-hexoside	17.60	354/303^sh^/255	463.8	301.2	UV/MS
5	quercetin-*O*-xilopyranoside	18.11	354/254	433.5	300.9	UV/MS
6	quercetin-*O*-pentoside	19.09	354/255	433.3	300.9	UV/MS
7	quercetin-*O*-pentoside	20.74	354/256	433.6	300.4	UV/MS
8	quercetin-*O*-deoxyhexoside	21.85	352/256	447.0	300.6	UV/MS
9	kaempferol-*O*-pentoside	23.07	361/263	417.2	285.4	UV/MS
10	luteolin-deoxyhexose	26.02	344/255	431.5	285.2	UV/MS
11	Quercetin	26.86	370/255	301.3	179,151, 137	UV/MS
12	Luteolin	29.08	350/253	285.3	–	UV/MS
13	Isorhamnetin	30.12	366/264	315.4	301.1	UV/MS

**Figure 1.**
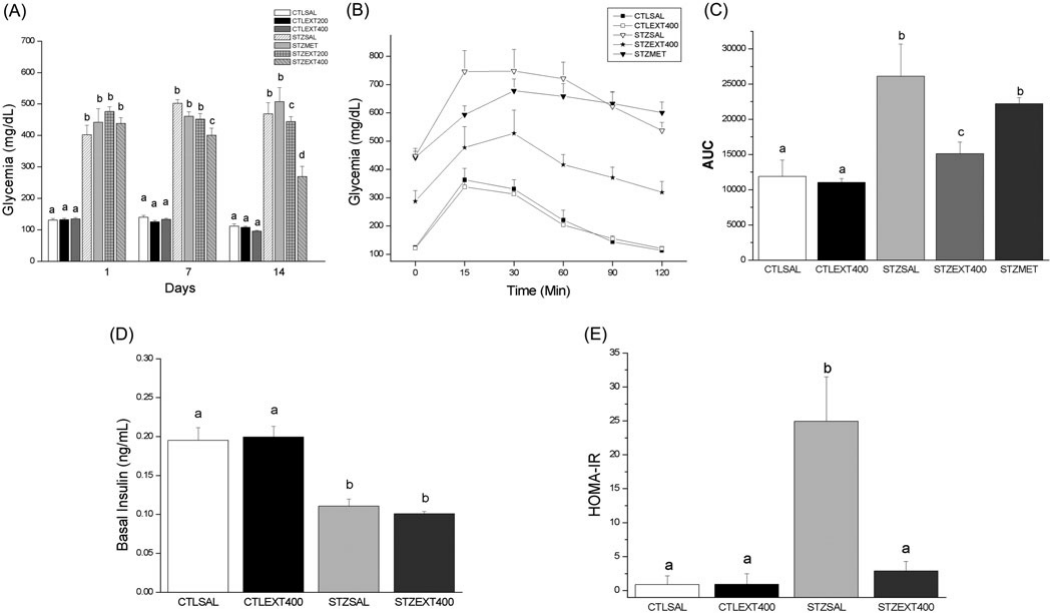
Effect of *Bauhinia holophylla* treatment on glycaemia, glucose tolerance and insulin sensitivity. (A) Average glycaemia during the treatment period. (B) Average glycaemia values during the intraperitoneal glucose tolerance test (ipGTT). (C) Areas under the curves (AUC) values obtained from ipGTT experiment. (D) Average insulinaemia at the end of the treatment period and (E) HOMA-IR index of the groups. *Bauhinia holophylla* treatment significantly decreased glycaemia and HOMA-IR index. Basal insulin values were not changed, suggesting an extra-pancreatic action of the extract. Different letters indicate significant differences (ANOVA followed by Tukeyʼs post-test, *n* = 8, *p* < 0.05).

Peak data correspond with peak numbers in Figure 1.

The chromatogram peaks with bands at *λ*_max_=330–380 nm and *λ*_max_=240–280 nm were typical of flavonoid derivatives of flavonols (Andersen and Markham 2006). Diagnostic mass fragments at *m/z* 301 and *m/z* 317 characterized quercetin and myricetin, respectively. Neutral losses of 132, 162 and 146 Da allowed the identification of pentosides, hexosides and deoxyhexosides, respectively. Multiple fragmentations of the parent ions were performed via FIA-ESI-IT-MS^n^. The ions at *m/z* 433 [M-H]^−^, *m/z* 447 [M-H]^−^, *m/z* 463 [M-H]^−^ and *m/z* 479 [M-H]^−^ were dereplicated as quercetin-*O*-pentoside, quercetin-*O*-deoxyhexoside, myricetin-*O*-hexoside and myricetin-*O*-hexoside, respectively.

### Acute toxicity test

Prior to the *in vivo* experiments, an acute toxicity test was performed in normoglycaemic mice (*n* = 10) administered a single dose of 2 g/kg b.w. of the extract (BH), while a control group received saline (CTL). No deaths or behavioural changes (posture, secretions or convulsions) were observed in the first 6 h after BH treatment, and no body weight differences occurred in the 14 days after treatment compared to those in the CTL group. The weights of internal organs (spleen, heart, liver, lungs and kidneys) were measured after 14 days, and no differences were observed between the two groups. According to these data, the extract was considered safe, even at this high dose.

### Glycaemia, effective dose and intraperitoneal ipGTT

Once diabetes had been established in the mouse model, all groups of normoglycaemic and diabetic mice were treated as appropriate for 14 consecutive days, and glycaemia was measured on days 0, 7 and 14. Treatment with 200 mg/kg b.w. of extract did not decrease fasting blood glucose levels in comparison with the levels in the STZSAL group. However, 400 mg/kg b.w. of extract produced a significant decrease in fasting blood glucose after the first week of treatment. By day 14, the STZEXT400 group showed a reduction of 50% in glucose levels compared with the levels in the STZSAL group (Figure 1(A), *p* < 0.01). Since the lower dose (200 mg/kg) had no effect on the fasting blood glucose, the higher dose of 400 mg/kg b.w. was used in subsequent experiments.

An improved glucose tolerance was observed in the STZEXT400 group compared with the STZSAL and STZMET groups in the ipGTT (Figure 1(B)). The STZEXT400 curve resembled that of the CTL groups, with a marked decrease in glycaemia from 30 min and a return to almost the baseline value at 120 min. However, the resulting area under the curve (AUC) indicated better glucose handling among diabetic mice treated with 400 mg/kg b.w. compared with the control mice (Figure 1(C)).

The capacity of flavonoids and other polyphenols to decrease blood glucose is well established (Kim et al. 2016), through multiple mechanisms in various organs (adipocytes, pancreatic β cells, liver, muscle and intestine). Abdelmoaty et al. (2010) showed that quercetin protected pancreatic islet cells against the oxidative stress caused by streptozotocin administration in rats, preventing hyperglycaemia and normalizing blood glucose levels. Ong and Khoo (2000) treated streptozotocin-diabetic rats with myricetin and observed reduced fasting blood glucose and increased glycogen content and glucose-6-phosphatase activity.

### Body weight, food and water intake

Apart from glycaemia, other parameters were improved following treatment with the *B. holophylla* extract. Compared with the CTLSAL group, the STZSAL group showed a significant (*p* < 0.05) decrease in body weight and an increase in food and water intake, which are characteristic symptoms of diabetes. Insulin exerts an anorexigenic action by binding to the insulin receptor (IR) and activating PI3K in the central nervous system (Niswender et al. 2003; Brown et al. 2006); in diabetic patients, the lack of insulin thus leads to an increase in food intake. However, the STZEXT400 group did not exhibit decreased body weight during the treatment period (Table 2), although food and water intake was decreased in the STZEXT400 group compared with the STZSAL group (Table 2, *p* < 0.01). Treatment with the *B. holophylla* extract was associated with an improvement in the primary diabetic symptoms as well as prevention of weight loss, indicating that the extract ameliorated the diabetic condition.

**Table 2.** Physical and biochemical parameters in various groups.

Parameters	CTLSAL	CTLEXT400	STZSAL	STZEXT400
Body weight (g) – Day 1	45.3 ± 1.2	44.6 ± 1.5	45.3 ± 1.5	46.1 ± 1.3
Body weight (g) – Day 14	47.0 ± 1.4^a^	47.5 ± 1.5^a^	35.0 ± 1.7^b^	43.1 ± 1.7^a^
Food intake (g/100 g bw.day)	18.9 ± 0.6^a^	16.7 ± 0.3^a^	35.7 ± 1.3^b^	27.5 ± 0.5^c^
Water intake (mL/animal.day)	11.8 ± 1.4^a^	8.7 ± 1.3^a^	60.7 ± 1.5^b^	47.2 ± 2.5^c^
Cholesterol (mg/dL)	89.6 ± 8.7^a^	93.8 ± 10.3^a^	103.0 ± 5.5^b^	72.7 ± 7.4^c^
Triglycerides (mg/dL)	107.1 ± 2.4^a^	106.9 ± 5.5^a^	138.9 ± 13.7^b^	90.5 ± 3.8^c^
Total proteins (g/dL)	5.3 ± 0.4^a^	5.6 ± 0.3^a^	5.3 ± 0.4^a^	5.6 ± 0.5^a^
Muscle glycogen (mg/%)	0.52 ± 0.06^a^	0.53 ± 0.03^a^	0.41 ± 0.03^b^	0.41 ± 0.02^b^
Hepatic glycogen (mg/%)	1.8 ± 0.2^a^	1.6 ± 0.3^a^	0.98 ± 0.1^b^	1.8 ± 0.2^c^

CTLSAL: Normoglycaemic mice treated with saline; CTLEXT: Normoglycaemic mice treated with *Bauhinia holophylla* extract (400 mg/kg); STZSAL: Diabetic mice treated with saline; STZEXT400: Diabetic mice treated with *B. holophylla* (400 mg/kg). Values are expressed as means ± SEM (*n* = 8/group). Different letters indicate significant differences (ANOVA followed by Tukeyʼs post-test, *p* < 0.05).

### Serum biochemical parameters, and hepatic and muscular glycogen levels

Lipid metabolism disturbances are common in diabetic patients due to the absence of insulin. In normal metabolism, insulin activates lipoprotein lipase, which hydrolyzes triglycerides. Insulin deficiency thus results in a lack of lipoprotein lipase activation, thereby causing hypertriglyceridaemia (Georg and Ludvik 2000; Rahman 2007). In the present study, diabetic mice in the STZSAL group exhibited a significant increase in cholesterol (TC) and triglyceride (TG) levels (*p* < 0.01), compared with the levels in the CTLSAL group. However, diabetic mice treated with the *B. holophylla* extract showed a significant reduction in TC and TGs (Table 2) compared with the STZSAL group. Interestingly, the treatment led to a significant reduction in these parameters, even when the diabetic treated group was compared with both control groups. This beneficial effect is consistent with the literature, which indicates that the intake of flavonoids or other phenolic compounds is associated with a decrease in cardiovascular diseases and atherosclerosis once these compounds are involved in lowering lipid levels, decreasing LDL oxidation, lowering blood pressure, and reducing inflammation and oxidative stress, thus improving the secondary complications of diabetes (Li et al. 2004).

Glucose is stored in the liver and muscle as glycogen through processes regulated by insulin, which is responsible for the stimulation of glycolysis and glycogenesis in these tissues as well as the inhibition of glycogenolysis and gluconeogenesis. In the present study, hepatic glycogen levels were markedly decreased in the STZSAL group compared with the CTLSAL and CTLEXT400 groups (*p* < 0.05), and treatment with the *B. holophylla* extract increased the hepatic glycogen content. Given that these processes are controlled by insulin and that treatment with the *B. holophylla* extract did not increase the basal insulinaemia (Figure 1(D)), these results indicate a possible insulin mimetic effect of the extract. Regarding muscle glycogen, the STZSAL and STZEXT400 groups presented similar decreases in glycogen content compared with the control (CTLSAL and CTLEXT400) groups, indicating that the liver is the main site of action of the *B. holophylla* extract (Table 2).

### Plasma insulin and HOMA-IR index

The decrease in fasting blood glucose and the improvement in glucose tolerance by the extract of *B. holophylla* raised some possibilities concerning its mechanism of action: the extract might directly induce insulin secretion by the pancreas, and/or the compounds present in the extract might exert an insulin-mimetic action on insulin-dependent processes. To clarify this point, the plasma insulin content was measured (Figure 1(D)) and the HOMA-IR index calculated (Figure 1(E)) after 14 days of treatment with the *B. holophylla* extract. Our results showed that, compared with the CTLSAL and CTLEXT400 groups, the STZSAL and STZEXT400 groups exhibited a decrease in plasma insulin, confirming the presence of diabetes. The administration of the *B. holophylla* extract did not alter the plasma insulin concentration, indicating an extra-pancreatic effect. The HOMA-IR index was significantly decreased in the STZEXT400 group compared with the STZSAL group, favouring an increase in the insulin pathway. Since the animals treated with *B. holophylla* had their insulin levels unchanged, but glucose levels significantly decrease, it is reasonable to assume that the decrease in glycaemia is due to increased sensitivity in peripherical tissues. This assumption showed to be correct, as can be seen by the results of protein expression experiments especially in the liver described below.

### Effect of B. holophylla treatment on the liver

#### Glycogenesis pathway stimulation

**Figure 2.**
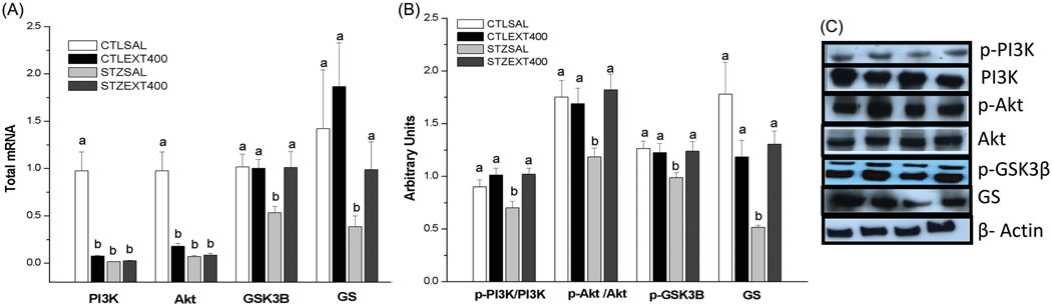
Liver glycogenesis stimulation by the treatment with *Bauhinia holophylla* extract. (A) The expression of *PI3K*, *Akt*, *GSK3-β* and *GS* genes analyzed by real-time PCR. (B) The expression of phosphorylated and total proteins by western blot. (C) Representative images of the proteins bands analyzed. Different letters indicate significant differences (ANOVA followed by Tukeyʼs post-test, *n* = 8, *p* < 0.05).

PI3K/Akt is the first pathway activated by insulin through the phosphorylation of its receptor (IR), resulting in the activation of a cascade of proteins that mediates the metabolic actions of insulin. PI3K, through its binding to phosphoinositide-dependent protein kinase (PDK), is responsible for the phosphorylation and activation of Akt, which is responsible for both the storage of glycogen and the inhibition of its degradation in peripheral tissues through glycogen synthase (GS) activation and glycogen phosphorylase inhibition, respectively (Chang et al. 2006; Choi et al. 2010). Our results showed that PI3K and Akt mRNA expression was decreased in the CTLEXT400, STZSAL and STZEXT400 groups compared with the CTLSAL group (Figure 2(A)). However, the mRNA expression of genes encoding GSK-3β and GS proteins was increased in the STZEXT400 group (almost 100 and 50%, respectively) compared with the STZSAL group. The expression of PI3K (p-PI3K), Akt (p-Akt), p-GSK3β (Ser-9) and GS proteins was increased in the STZEXT400 group by 53, 43, 25 and 153%, respectively, compared with the expression in the STZSAL group (Figure 2(B,C)). Liver GS is regulated by phosphorylation at multiple sites. GSK-3β, a Ser/Thr kinase, phosphorylates and inhibits GS. Insulin, through the activation of the PI3K/Akt pathway, inhibits GSK-3β by the phosphorylation of Ser-9 residues, thus preventing the inhibition of GS and resulting in increased glycogenesis (Stambolic and Woodgett 1994). Both GS and GSK-3β are highly complex enzymes in terms of function and regulation. Hepatic GS can be inactivated by phosphorylation at different sites by different kinases (Pugazhenthi and Khandelwal 1995), and GSK-3β phosphorylates diverse substrates and is involved in several pathophysiological processes (Rayasam et al. 2009). In the liver, GSK-3β is the most potent inhibitor of GS and, consequently, of glycogenesis and therefore represents a promising target for diabetes treatment (Coghlan et al. 2000; Cline et al. 2002). Our results show that the glucose-lowering effect exerted by a *B. holophylla* extract may be attributed, at least in part, to the stimulation of glycogenesis. In a previous study by our research group, Vareda et al. (2014) reported that the mechanism for the observed hypoglycaemic effect of a crude extract of *Myrcia bella* Cambess. (Myrtaceae) involves the modulation of the expression of important proteins responsible for glycogen storage, indicating that this process may be modulated by medicinal plants to improve the control of glycaemia.

In contrast, a recent study by Pinheiro et al. (2017) showed that treatment with an aqueous extract of the leaves of *B. holophylla* at 400 mg/kg did not decrease glycaemia in either diabetic or non-diabetic rats. These opposing results may be due to the absence of active substances in the boiled aqueous extract used or to the misidentification of *B. holophylla*, given the previously described complexity of this task, as highlighted by several other research groups (Queiroz 2006; Vaz 2010).

#### Gluconeogenesis inhibition

**Figure 3.**
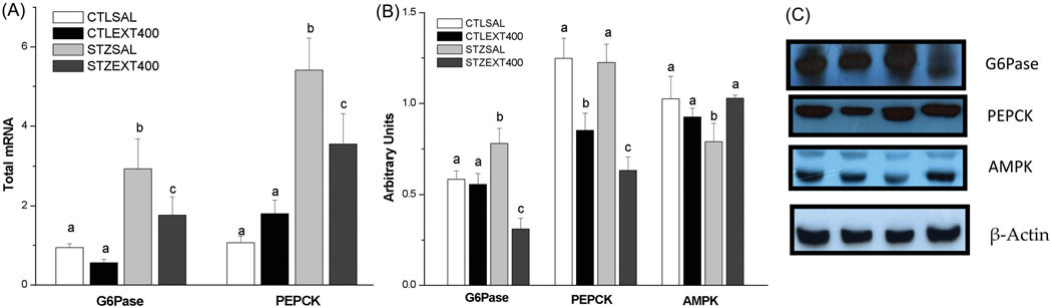
Liver gluconeogenesis inhibition by the treatment with *Bauhinia holophylla.* (A) *G6Pase* and *PEPCK* genes expression analyzed by real-time PCR. (B) Protein expression of G6Pase, PEPCK and AMPK analyzed by western blot. (C) Representative images of the bands. Different letters indicate significant differences (ANOVA followed by Tukeyʼs post-test, *n* = 8, *p* < 0.05).

During fasting periods, ∼85% of endogenous glucose produced from the liver is derived from glycogenolysis and gluconeogenesis. As fasting continues, gluconeogenesis becomes increasingly important in the maintenance of blood glucose. Glucose-6-phosphate (G6Pase) and phosphoenolpyruvate carboxykinase (PEPCK) are key gluconeogenesis enzymes that are inhibited by insulin in the postprandial period (van Schaftingen and Gerin 2002; Estrada et al. 2005). As a result of insulin deficiency and increased glucagon in diabetes, these enzymes are increased, leading to a stimulation of gluconeogenesis that is critical for the hyperglycaemic state (Collins et al. 2006). AMPK is an energy-sensing enzyme activated by an elevation of the cellular AMP:ATP ratio. AMPK activates PI3K/Akt in an insulin-independent manner and, together with Akt, inhibits G6Pase and PEPCK, acting as a powerful inhibitor of gluconeogenesis (Hardie et al. 2012). Our results showed that the gene and protein expression of G6Pase and PEPCK (Figure 3(A–C)) were significantly decreased, whereas the expression of AMPK protein was increased (Figure 3(B,C)) in the STZEXT400 liver compared with the STZSAL liver, indicating the inhibition of gluconeogenesis and a subsequent decrease in hyperglycaemia.

Given that an elevated hepatic glucose output is considered the major abnormality associated with elevated plasma glucose (DeFronzo 2004), by stimulating glycogenesis in parallel with inhibiting gluconeogenesis, the *B. holophylla* extract represents an interesting and promising material for further investigation.

#### Effect of B. holophylla treatment on muscle

##### PI3K/Akt pathway activation

Beyond the liver, muscle is an important tissue in glucose homeostasis. Glucose entry into muscle cells is mediated by two pathways: PI3K/Akt (insulin dependent) and muscle contraction (AMPK stimulated, insulin independent). The first of these pathways commences with the binding of insulin to its receptor (IR), leading to the activation (phosphorylation) of a cascade of proteins such as IRS, PI3K, Akt and, finally, glucose transporter 4 (GLUT4). The increase in the AMP:ATP cellular ratio caused by muscle contraction activates AMPK, which activates multiple kinases such as ERK1/2 or AS160, resulting in the translocation of GLUT4 from the intracellular vesicles to the plasma membrane (Hawley and Lessard 2008).

**Figure 4.**
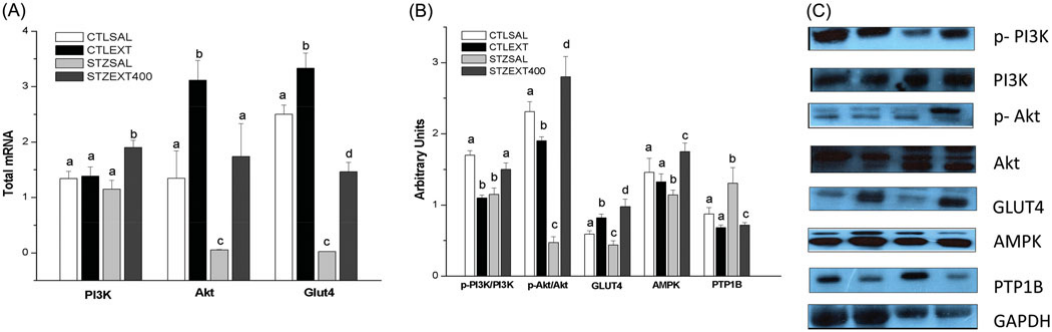
Effect of *Bauhinia holophylla* treatment on gene and protein expression in muscle. (A) Expression of *PI3K*, *Akt* and *Glut-4* genes. (B) Expression of phosphorylated and total forms of proteins involved in glucose uptake in muscles. (C) Representative images of the proteins analyzed. Different letters indicate significant differences (ANOVA followed by Tukeyʼs post-test, *n* = 8, *p* < 0.05).

In the present work, muscle tissue in the STZSAL group showed decreased expression of Akt and GLUT4 genes (Figure 4(A)) and proteins (Figure 4(B,C)). However, the diabetic group treated with the extract of *B. holophylla* showed increased expression of these genes and proteins, which likely contributed to the decrease in glycaemia through the increase in GLUT4 expression following activation of the PI3K/Akt pathway. Moreover, AMPK expression in muscle (Figure 4(B,C)) was increased in the STZEXT400 group compared with the STZSAL group, which contributed to the increase in GLUT4 expression and subsequent decrease in blood glucose – an effect demonstrated in recent studies (Moradabadi et al. 2013; Nikzamir et al. 2014).

##### PTP1B protein expression

Members of the tyrosine phosphatase family (PTPs), in which PTP1B occupies a prominent position, are negative regulators of IR. Therefore, the identification of PTP1B inhibitors is of therapeutic interest as targets for insulin resistance and diabetes (Gonzales-Rodrigues et al. 2010; Bakke and Haj 2015).

Some studies have demonstrated a PTP1B inhibitory effect of medicinal plants (Chen et al. 2002; Jiang et al. 2012). The compounds isolated from *Selaginella tamariscina* (P. Beauv.) Spring (Selaginellaceae) and *Camellia japonica* Champ. (Theaceae), for example, show a potent inhibitory effect on PTP1B enzyme activity and therefore represent potential candidates for development as antidiabetic agents (Uddin et al. 2014; Nguyen et al. 2015). In our study, the PTP1B protein expression decreased following treatment with the *B. holophylla* extract (Figure 4(B,C)) in the STZEXT400 group compared with the STZSAL group, leading to activation of the insulin signalling pathway as described above.

##### *In vitro* and *in vivo* α-amylase and α-glucosidase inhibition

α-Amylase and α-glucosidase are enzymes involved in carbohydrate metabolism. α-Amylase degrades complex carbohydrates to oligosaccharides and disaccharides, which are subsequently converted into monosaccharides by α-glucosidase; liberated glucose is then absorbed by the gut, increasing postprandial glucose levels. For this reason, inhibitors of these enzymes limit postprandial glucose levels by delaying glucose breakdown and absorption (Shihabudeen et al. 2011; Rajasekar et al. 2014).

**Figure 5.**
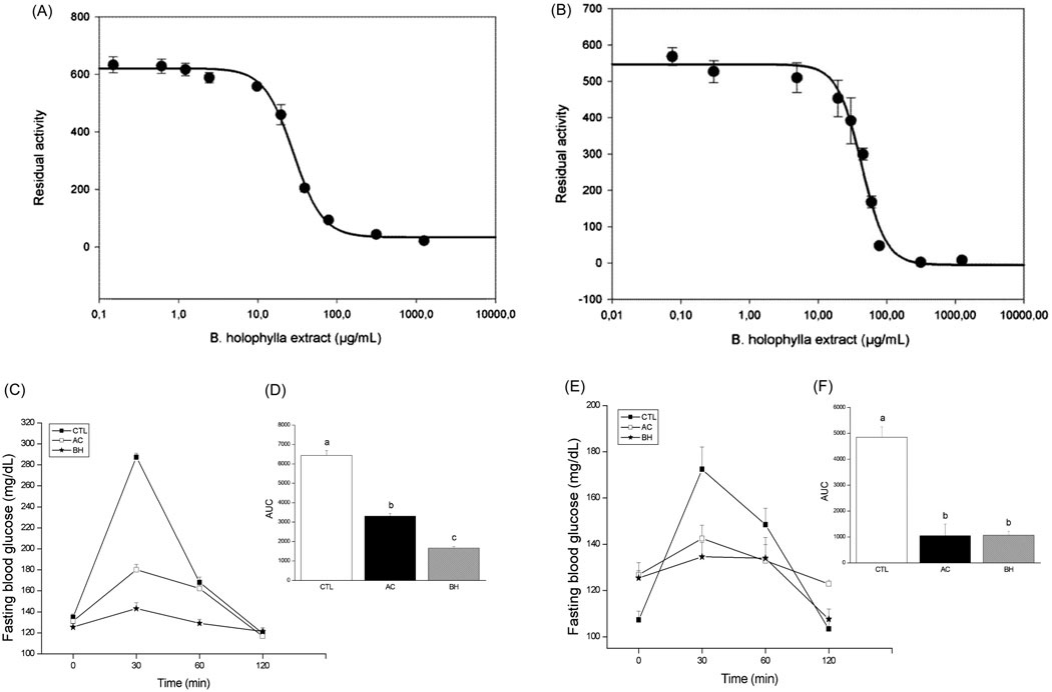
The inhibitory effect of the *Bauhinia holophylla* treatment on intestinal enzymes. *In vitro* determination of the IC_50_ for α-amylase (A) and α-glucosidase (B). *In vivo* inhibition of α-amylase after an oral starch load (C) with the respective areas under the curves (D) and of α-glucosidase (E) with the respective areas under the curves (F). Different letters indicate significant differences (ANOVA followed by Tukeyʼs post-test, *n* = 6, *p* < 0.05).

To investigate the mechanisms of action of the crude extract of *B. holophylla*, we evaluated its potential inhibitory activity in two commercial enzyme assays of digestive α-glucosidases: pancreatic α-amylase and yeast maltase. The IC_50_ and binding mode (reversible or irreversible) were determined. The results demonstrated that crude extracts of *B. holophylla* showed an IC_50_ of 28.0 μg/mL (Figure 5(A)) for α-amylase, and 43.0 μg/mL (Figure 5(B)) for α-glucosidase, indicating its increased potency against α-amylase. Acarbose has been shown to have an IC_50_ of 2.7 μg/mL for amylase and an IC_50_ of 516.1 μg/mL for maltase (Andrade-Cetto et al. 2008; Senger et al. 2012; Wang et al. 2016). Although the IC_50_ of the *B. holophylla* extract was higher than that of acarbose for α-amylase, it must be considered that our study utilized a purified compound compared with a crude extract and that the *B. holophylla* extract was over 100 times more efficient in inhibiting maltase compared with acarbose. The extract appeared to interact with both glucosidases in a slow, reversible mode, as the progress curves were linear with the slope displaced to the right (data not shown).

Following the promising *in vitro* inhibitory results of the extract, we performed an *in vivo* assay in a mouse model of diabetes. To evaluate the potential α-amylase inhibitory effect, normoglycaemic mice treated with *B. holophylla* (400 mg/kg b.w.) received a solution of starch, and the blood glucose variation was measured. Our results showed that the control group had an extremely high level of blood glucose 15 min after starch and maltose loading, demonstrating the breakdown and absorption of starch as glucose. However, the co-administration of starch and *B. holophylla* at a dose of 400 mg/kg prevented the peak in glucose following starch administration (Figure 5(C,D)). In the α-glucosidase assay, normoglycaemic mice treated with *B. holophylla* (400 mg/kg b.w.) received a solution of maltose, and the blood glucose variation was then measured (Figure 5(E,F)). Control group mice treated with saline showed an increase in glucose levels following the administration of the maltose solution, indicating the complete breakdown and absorption of monosaccharides from maltose. In contrast, the BH group showed a lower glucose peak following maltose administration, indicating that the breakdown and absorption of monosaccharides from maltose was inhibited. Acarbose was used as a reference standard for evaluating the α-amylase inhibitory action. The crude *B. holophylla* extract exhibited appreciable α-glucosidase inhibitory effects compared with acarbose. Medicinal plants with α-glucosidase inhibitor effects have been suggested to represent a prospective therapeutic approach to the management of postprandial hyperglycaemia (Rios et al. 2015).

## Conclusions

The qualitative and quantitative analysis of the chemical profile of a 70% hydroethanolic extract of *B. holophylla* revealed the presence of flavonoid-*O*-glycosides derivatives, mainly flavonols. *In vivo* testing showed that the hypoglycaemic activity of the *B. holophylla* extract occurred by modulation of insulin-dependent processes such as the stimulation of liver glycogenesis by inhibition of GSK3-β through PI3K/Akt pathway and the inhibition of gluconeogenesis, as well as by increasing the expression of proteins involved in glucose uptake in the muscle without increasing basal insulin levels, thus indicating an extra-pancreatic mechanism of action. Moreover, the extract was shown to function as a hypolipidaemic agent by decreasing serum lipid levels and, importantly, also showed the ability to inhibit intestinal α-glucosidase enzymes – a key effect in the impairment of postprandial hyperglycaemia. The characterization of a crude extract of *B. holophylla* as a hypoglycaemic and hypolipidaemic agent represents an interesting pathway for further investigation to better understand the mechanism of action of the extract.
